# Anthropogenic influence on the changing risk of heat waves over India

**DOI:** 10.1038/s41598-022-07373-3

**Published:** 2022-02-28

**Authors:** P. Kishore, Ghouse Basha, M. Venkat Ratnam, Amir AghaKouchak, Qiaohong Sun, Isabella Velicogna, T. B. J. M. Ouarda

**Affiliations:** 1 Department of Earth System Science, University of California, Irvine, CA 92697 USA; 2 Department of Space, National Atmospheric Research Laboratory, Gadanki, P.B. No123, Tirupati, 517502 India; 3 Department of Civil and Environmental Engineering, University of California, Irvine, CA 92697 USA; 4 Jet Propulsion Laboratory, California Institute of Technology, Pasadena, CA 91109 USA; 5 INRS-ETE, National Institute of Scientific Research, Quebec City, Canada

**Keywords:** Climate sciences, Environmental sciences

## Abstract

The overarching goal of this paper is to shed light on the human influence on the changing patterns of heat waves in India using the Heat Wave Magnitude Index daily (HWMId). The HWMId obtained from the observational data sets shows a large increase in the heat waves during the past decades. Investigating the effects of natural (e.g., solar variations and volcanic forcings) and anthropogenic (e.g., greenhouse gas emissions, anthropogenic, land use, and land cover) forcings revealed that the anthropogenic factors have cause a two-fold increase in the occurrence probability of severe heat waves in central and mid-southern India during twentieth century. The spatial distribution of maximum HWMId values under natural and all forcings (including anthropogenic) indicates that in most places human activities have increases the frequency, duration and intensity of extreme heat waves. Under the Representative Concentration Pathway (RCP) 4.5, the risk of heat waves is projected to increase tenfold during the twenty-first century. More than ~ 70% of the land areas in India is projected to be influenced by heat waves with magnitudes greater than 9. Furthermore, we find a significant relationship between heat waves and deficits in precipitation. Results show that concurrent heat waves and droughts are projected to increase in most places in India during the twenty-first century.

## Introduction

A heat wave is generally defined as a prolonged period of excessively hot weather. Prolonged and intense heat waves have become more frequent in many parts of the globe^1^. This leads to short-term increases in mortality and negative impacts on infrastructure and on biophysical systems^2^. The annual mean temperature over India has increased by 0.85 °C from 1901 to 2015^3^. Based on climate model simulations, the surface temperature is expected to rise substantially by the end of this century^3^. Increasing mean temperatures lead to more intense heat waves that last longer and/or occur more frequently^1,4^. Several studies have showed evidence of change in the frequency and duration of heat waves over India and discussed the underlying mechanisms^5,6^. Under a 2 °C mean temperature increase experiment, the frequency of heat waves is expected to rise by 2.5 times by the end of the twenty-first century^7^.

While many studies have investigated anthropogenic influences on the frequency and/or severity of heatwaves at the global scale^8–10^, we are not aware of any India-specific study on natural and human influences on the changing patterns of heat waves using the Heat Wave Magnitude Index daily (HWMId) (see the “Methodology” section for details of HWMId estimation). Therefore, the main objective of the present study is to understand the influence of the Natural (NAT–e.g., solar variations and volcanic forcings) and Anthropogenic (e.g., greenhouse gas emissions, anthropogenic, land use, and land cover) forcings on the occurrence of heat waves as defined with HWMId. The future risk ratio during the historical period relative to the NAT forcings are estimated under the Representative Concentration Pathways (RCP) 2.6, 4.5, 6.0 and 8.0 scenarios during twenty-first century. Finally, we discuss cooccurrence of heat waves and droughts based on the Standard Precipitation Index (SPI).

## Results and discussion

**Figure 1:**
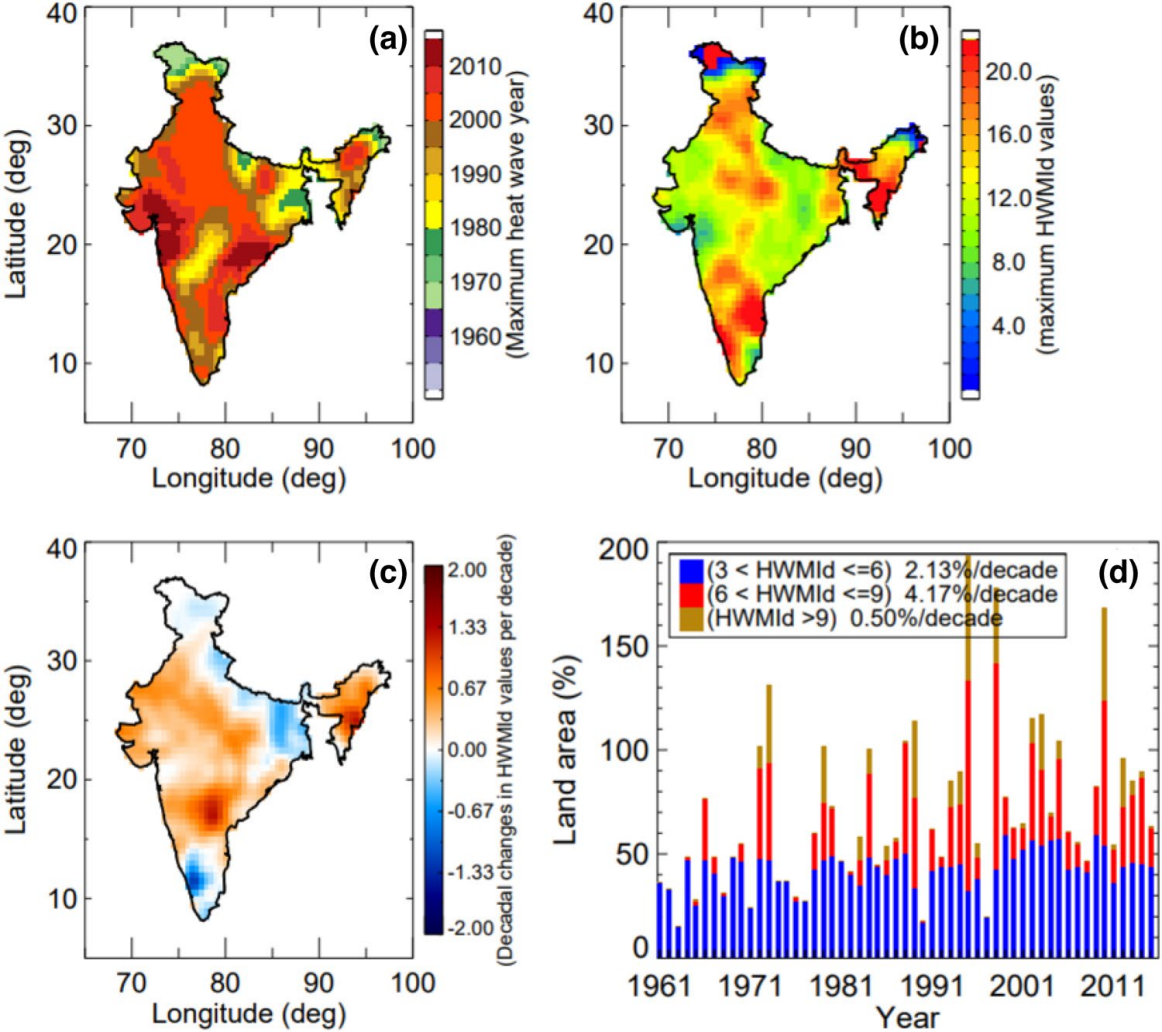
Spatial distribution of the observed Heat Wave Magnitude Index daily (HWMId) values estimated using IMD gridded data from 1961–2015. (**a**) Maximum heat waves occurred during the year based on HWMId values. (**b**) Maximum observed HWMId values. (**c**) Changes in HWMId values per decade. (**d**) Time series of percentage of the land area affected by heat waves over India (3 < HWMId ≤ 6; 6 < HWMId ≤ 9; HWMId > 9). (Figure was created using the Interactive Data Language (IDL) version 8.2 software, http://www.harrisgeospatial.com/docs/whats_new_in_82.html).

Figure 1 shows the distribution of the observed Heat Wave Magnitude Index daily (HWMId) values estimated based on the gridded gauge data from the India Meteorological Department (IMD) during 1961–2015. Most extreme heat waves occurred during 2000 to 2015 in many parts of India (Fig. 1a), particularly the East Coast, North West, and central India. In recent decades, the East Coast (Orissa and Andhra Pradesh) and some parts of the West Coast (Mumbai and Gujarat) have been significantly affected by heat waves. These regions were heavily affected during the 2015 heat wave which resulted in more than ~ 2500 human deaths in a span of a few days^11–13^*.* Most of the country has experienced HWMId values larger than 3 and a significant part values larger than 5 (Fig. 1b). This signifies the occurrence of heat waves are large throughout India. The HWMId values show positive trends in 80% of the grid cells (Fig. 1c). Significant changes are noted over Gujarat, Rajasthan, and the North East region of India. Negative trends are observed over West Bengal and Kerala (Fig. 1c). Due to increases in temperature, a large portion of the land now suffers from more frequent and intense heat waves. In particular, Fig. 1d shows significant increases in the areas affected by heat waves at different magnitudes (3 < HWMId ≤ 6, 6 < HWMId ≤ 9, HWMId > 9). The region affected by HWMId > 9 heat waves is significantly larger than those affected by other magnitudes of heat waves (Fig. 1d). Note that the area affected by heat waves expanded by threefold in the period 2000–2015 as compared to previous years. Furthermore, the land area was affected by HWMId > 9 heat waves during the period 1995–2005 (Fig. 1d) is large compared to previous years. Before investigating heat waves based on the Coupled Model Intercomparison Project Phase 5 (CMIP5) under historical (including natural and anthropogenic) and natural only (NAT), we evaluated the consistency of the historical simulations with observations (see Fig. S1). Both data sets illustrate a similar distribution of heat waves spatially across India.

**Figure 2:**
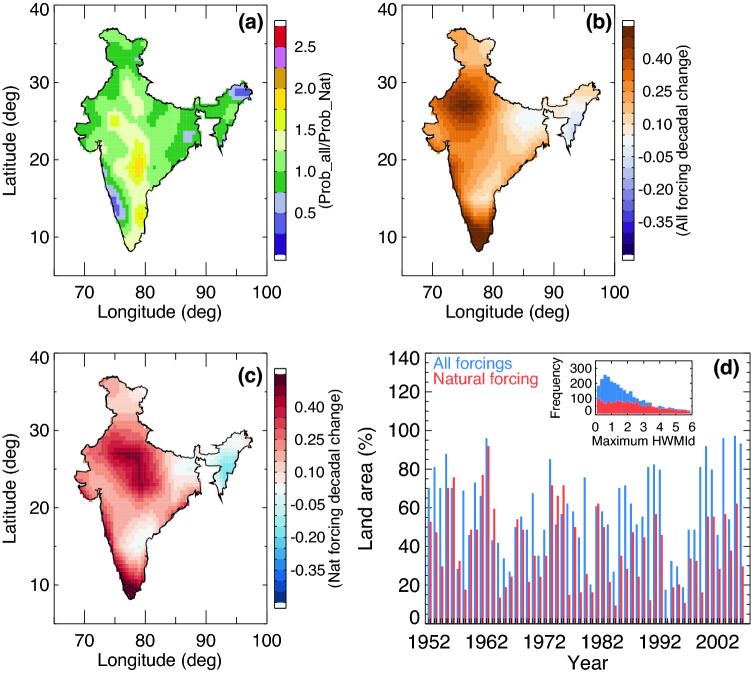
Spatial distribution of simulated Heat Wave Magnitude Index daily (HWMId) values from natural forcing (NAT) and all forcings (historical). (**a**) Risk ratio for each grid location  from natural and all forcings during the period 1961–2005. Decadal changes in HWMId values in all forcings (**b**) and natural forcings (**c**) during the period 1961–2005 (absolute changes in HWMId values per decade). (**d**) Percentage of land area affected by heat waves with HWMId > 1 from natural and all forcings. Inset: Histogram of HWMId values from natural and all forcings. (Figure was created using the Interactive Data Language (IDL) version 8.2 software, http://www.harrisgeospatial.com/docs/whats_new_in_82.html).

We then compared the probability of maximum HWMId values from all forcings to NAT forcing for each grid location using the so-called risk ratio [see Eq. (2) in “Methodology” section]. Large values (> 2.5) of risk ratio signify strong increases in heat waves when anthropogenic influences are included. Over central and mid-southern India, the anthropogenic factors cause a two-fold increase in the occurrence probability of severe heat waves (Fig. 2a). The decadal changes in HWMId values provides further evidence that the increase in heat waves is primarily dominated by anthropogenic factors (compare Fig. 2b,c). The increase in anthropogenic influences has led the Indian region to experience a greater number of heat waves, particularly in the recent past (Fig. 2d). The deadly heat wave during 2015^12^ was among one of the most devastating events in the recent past. The influence of anthropogenic forcing is significantly larger compared to NAT forcing. More than ~ 70% of India’s landmass experiences heat waves when all the forcings are included in the simulations. The distribution of maximum HWMId values (Fig. 2d, inset) shows the peak is larger under all forcings compared to NAT forcing alone, indicating that human influences over India caused the increase in frequency, duration and intensity of extreme heat waves.

**Figure 3:**
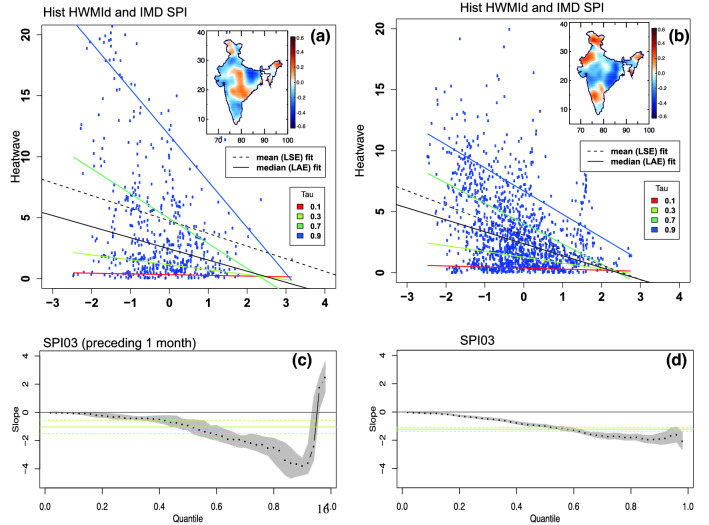
Quantile regression analysis between heat waves and 3-month standardized precipitation index (SPI03) using data from all grids. Scatter plots of the maximum HWMId values for each grid location over India during the period 1961–2015 and the 3-month SPI preceding 1 month (**a**) and concurrent month (**b**). Inset figures display the spatial correlation maps of HWMId- SPI03. Panels (**c**) and (**d**) show the corresponding regression slopes for 0.1–0.99 quantiles of the maximum HWMId-SPI03. Black filled circles indicate the regression slope for different quantiles and shading indicates the associated uncertainty. The horizontal solid line represents the mean trend based on the ordinary least-squares method. (Figure was created using the Interactive Data Language (IDL) version 8.2 software, http://www.harrisgeospatial.com/docs/whats_new_in_82.html).

We used quantile regression analysis to investigate the relation between 3-month SPI (dry/wet conditions) and HWMId values (lagged by 1 month) at different quantiles (10 to 99%). The upper (bottom) panel of Fig. 3 shows the scatter plot (quantile regression) results between HWMId and 3-month SPI. Results indicate that more heat waves occur during dry conditions (i.e., negative SPI events). Figure 3c,d summarize the quantile regression results across different quantiles highlighting the negative regression slopes between HWMId-SPI (for similar quantile regression analysis but for SPI values lagged by + 1 month see Fig. S2 in Supplementary Information).

**Figure 4:**
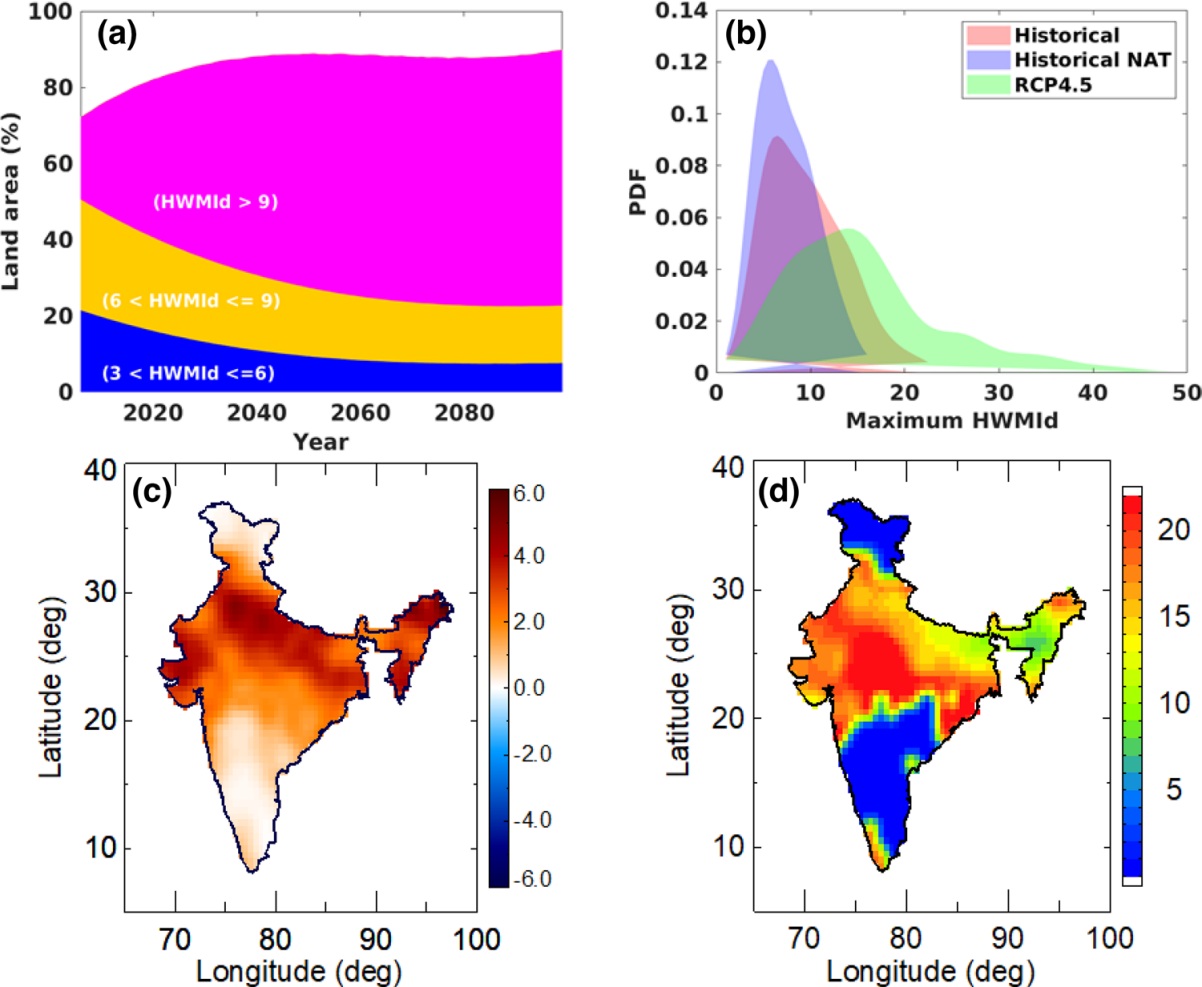
Spatial distribution of projected HWMId values under RCP4.5. (**a**) Percentage of land area affected by different heat wave severity levels (3 < HWMId ≤ 6; 6 < HWMId ≤ 9; HWMId > 9) under RCP4.5 for the period 2006–2099. (**b**) Maximum HWMId probability density functions estimated from each grid location from natural (1961–2005), Historical (1961–2005) and RCP4.5 (2045–2099) experiments. (**c**) Spatial distribution of decadal changes in absolute risk ratio calculated for each grid using a 55-year moving window over the period 2006–2099 from RCP4.5 scenario. (**d**) Spatial differences between maximum HWMId risk ratio for the projection period (2045–2099) relative to the historical period (1961–2005) under RCP4.5 (figure was created using the Interactive Data Language (IDL) version 8.2 software, http://www.harrisgeospatial.com/docs/whats_new_in_82.html).

To investigate changes in future heat waves, we considered climate model simulations under RCP2.6, RCP4.5, RCP6 and RCP8.5. Results for RCP4.5 are presented in the paper whereas other RCPs are presented in Supplementary Information. Under the RCP4.5 scenario, the land area affected by heat waves over India is projected to increase during the period 2006–2099 when HWMId > 9 (Fig. 4a). The total land area affected by heatwaves( 3 < HWMId ≤ 6 and 6 < HWMId ≤ 6) is projected to decrease whereas area affected by heat waves with magnitude (HWMId > 9) is expected to increase by the end of the twenty-first century (Fig. 4a). The probability distribution functions (PDFs) of HWMId values for historical, NAT and future projections indicate not only anthropogenic forcings have shifted the heat waves PDFs toward more extreme events but also more intense changes are expected in a warming climate (similar results are presented for other RCPs in Figs. S3b, S4b, and S5b). This indicates an increased probability of extreme (HWMId > 6) and very extreme (HWMId > 9) heat waves in the future. Further, we estimate changes to the risk ratio over the years. We first computed a moving average window of 55-years to obtain a total of 30 risk ratio for the period 2006–2099 (55-year time periods, for 2016–2060, 2017–2061…,2044–2098, 2045–2099). We then calculated the risk of future heat wave for each 55-year period and estimated the rate of change of the risk ratio at each grid. The risk ratio is expected to increase drastically with anthropogenic warming in all the coastal and western parts of India and over various other locations (Fig. 4c). Spatial differences between maximum HWMId risk ratio for the projection period (2045–2099) relative to the historical period (1961–2005) under RCP4.5 reveals 5- to 20-fold increase in the occurrence likelihood extreme heat waves (Fig. 4d). The projected increases are more pronounced under RCP8.5 (see Fig. S5). Figures S3, S4, and S5 in Supplementary Information present the same results as Fig. 4 but for RCP2.6, RCP6 and RCP8.5, respectively.

**Figure 5:**
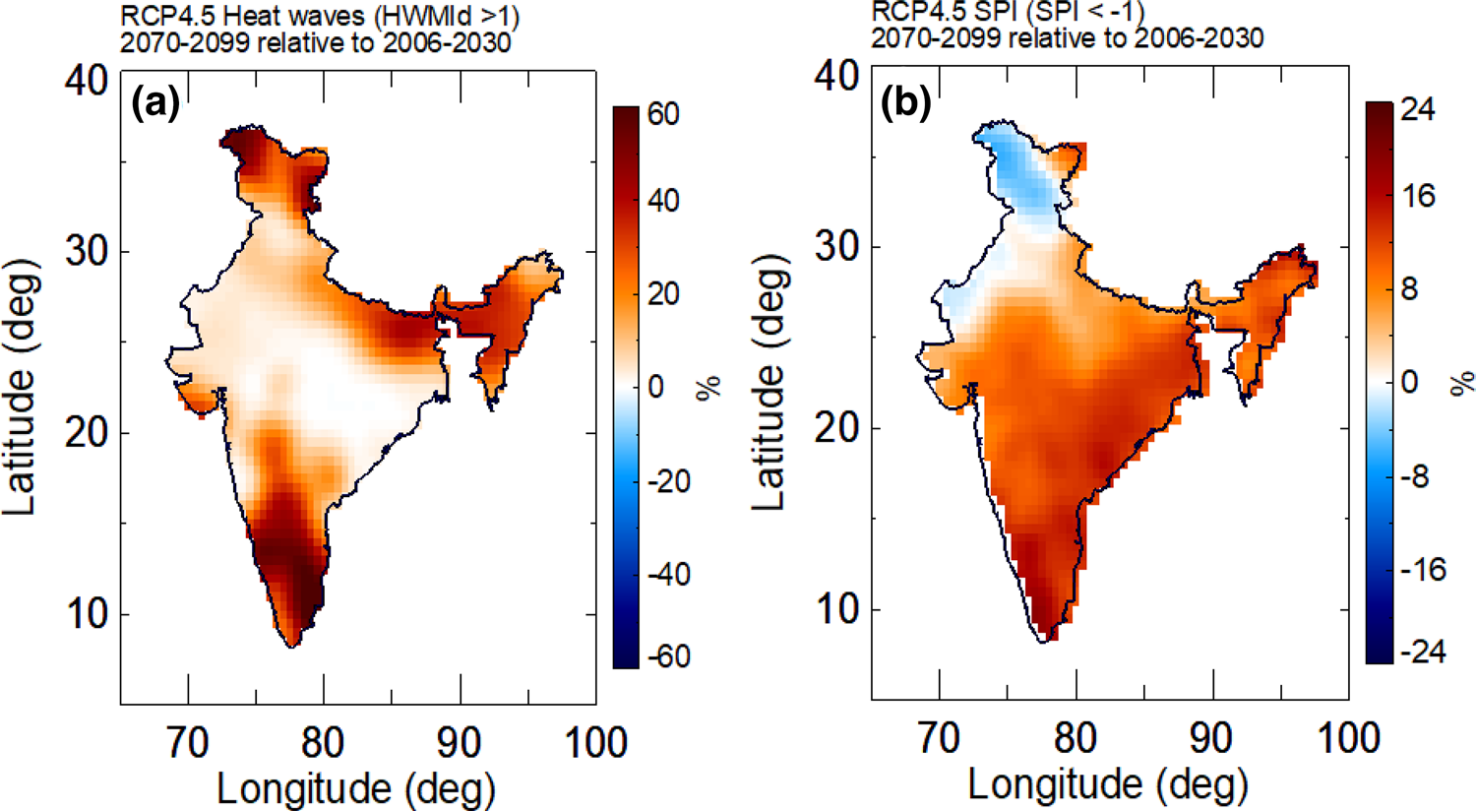
Spatial change in the frequency of concurrent heat waves (HWMId ≥ 1) and drought (3-month SPI ≤ − 1) under RCP4.5 during 2070–2099 relative to 2006–2030. (**a**) Heat waves occurring one month after drought. (**b**) Heat waves occurring simultaneously with drought (figure was created using the Interactive Data Language (IDL) version 8.2 software, http://www.harrisgeospatial.com/docs/whats_new_in_82.html).

Understanding the interaction between the occurrence of heat waves and droughts is important for more accurate assessment of heat waves and their potential to become human disasters. Heat waves are projected to follow droughts in many parts of India (Figs. 5a and S6) under different RCP scenarios. Mishra et al. reported that frequency of heat waves will rise by 30 times by the end of twenty-first century if we do not take the necessary actions to limit the Earth’s warming^7^. The probability of these extreme events is projected to increase significantly over the western and northern parts of India with a higher frequency of concurrent droughts and heat waves in the near future (2077–2099) relative to 2006–2030 (Fig. 5b).

## Conclusions

Given the adverse effects of heat waves, understanding their variability and change are very important for densely populations regions with fragile ecosystems, such as India. In this study, we examined the occurrence of heat waves in India during the twentieth and twenty-first centuries using CMIP5 climate model simulations. The factors responsible for increases in heat waves were also studied with the help of NAT and anthropogenic forcing and historical observation through detection and attribution analysis. Observational data reveals a significant increase in land areas in India affected by intense heat in recent decades. Our attribution analysis indicates that heat wave frequency and intensity drastically increase in India during the twentieth century due to anthropogenic forcings. These heat waves are resulted in significant risk and detrimental effects on human health. Central India and major parts of the eastern coast experienced deadly heat waves during 2015 that were likely intensified due to anthropogenic climate change.

This increased risk in the occurrence of heat waves is evident in the reported Heat Wave Magnitude Index (HWMId) values and their temporal variability. Our analysis shows that the land areas affected by heat waves with magnitude HWMId > 9 are projected to increase significantly compared to lower magnitudes by the end of the twenty-first century. Model simulations indicate occurrence of unprecedented heat waves in the future never observed in the observational period (e.g., see Figs. 4, S3, S4 and S5)^4^. Furthermore, we quantify the relationship between heat waves and SPI (for dry and wet conditions). Previous studies have shown that dry conditions affect the evolution of heat waves all over the world^12,13^. Here we observed a negative correlation between SPI and heat waves through the use of quantile regression analysis. Drier conditions are associated with stronger heat waves throughout India. Future increases in summer dry conditions will most likely contribute to widespread, long-lasting, severe heat waves across India. Considering the observed and projected changes in heat waves along with population and industrial growth, puts substantial pressure on the environment, human health, agriculture, and energy sector if adaption and mitigation measures are not put in place.

## Data and methods

We used India Meteorological Department (IMD) surface temperature data available at a 1° × 1° gridded for the period 1961 to 2015^14^. A total of 395 stations were gridded using a modified version of the Shepard angular distance weighting algorithm after applying quality checks^15^.

Long-term historical simulations from CMIP5 are used in this study^16^. The historical data spans the period 1850–2005, which is forced by observed atmospheric composition changes (including GHGs, natural and anthropogenic aerosols, and volcanic forcings), solar variations, land use, and land cover to simulate the observed climate of the recent historical period. Table S1 shows the CMIP5 models (Historical, NAT forcing and RCPs) used in the present study. An earlier study investigating the performance of 7 CMIP5 models against IMD gridded temperature and Climate Research Unit (CRU) data found that only 8 models perform well over India^3^. The same CMIP5 models (i.e., CNRM-CM5, CanESM2, GFDL-CM3, IPSL-CM5A-LR, MIRCOC5, MPI-ESM-LR, NorESM1-M, and bcc-csm1-1) are used in the present study. The selected CMIP5 models have been averaged for all historical and future projections of and used for estimating the HWMId. The same models from all RCPs were also considered for estimating HWMId projections in a warming climate.

## Methodology

Heat waves can be defined differently. Here, the notion of HWMId is used to quantitatively define duration and intensity of heat waves. HWMId denotes the number of heat waves with durations ≥ 3 consecutive days above a defined temperature threshold. In this study, a heat wave is defined as three or more consecutive days with daily maximum temperature higher than the 90th percentile of 30 or 31-day (depending on the month) running windows during the baseline period (1961–1990). This window size produces a reasonable sample size to calculate meaningful percentile values^17^. Heat waves are then classified into weak (1 < HWMId < 2), Moderate (2 ≤ HWMId < 3) and Intense (HWMId ≥ 3). The HWMId estimation procedure is available in the ‘extRemes’ R-package (open source) code.

The robust regression technique is used for estimating the trends and decadal changes based on Iteratively Reweighted Least Squares Regression^18^. The t-test analysis is used to estimate the statistical significance of the decadal trends throughout this paper at 0.05 significance level (e.g., Figs. 1c, 2b,c, and 4c)—i.e., only statistically significant trend values are considered.

### Attribution analysis

We used the risk ratio concept for attribution of heat waves. To determine the influence of climate change on the risk of heat waves, the probability of the maximum heat wave occurring under an all-forcings scenario vs. a natural forcing scenario is estimated and compared. The contribution of anthropogenic influence of climate changes to the risk of heat waves is estimated as follows:

1 $$Risk \; Ratio=\frac{{P}_{all}}{{P}_{nat}}$$

where P_nat_ and P_all_ are the probabilities of the observed events occurring under the NAT forcing and all forcings, respectively. This index compares the probability of extreme events occurring between the real world (including human influences) relative to that of the natural world without human influence.

A previous study^19^ reported the greatest warming in the second half of the twentieth century in the Northern Hemisphere and found it to be warmer than in any other 50-year period in the last 500 years. So, we opted for a 55-year moving window for the period of 2016–2099, which yields a total of thirty 55-year time periods (e.g., 2016–2060, 2017–2061,…2044–2098, 2045–2099) to estimate heat wave risk ratio for each 55-year period at each grid location during twenty-first century.

Estimation of probability ratios and risk ratio (e.g., Figs. 2a and 4d) rely on fitting the Generalized Extreme Value (GEV^20^) distribution to HWMId. The GEV distribution provides insights into the behavior of extremes and their reoccurrence intervals^21,22^. The goodness-of-fit was verified using the Kolmogorov–Smirnov (K-S) test at 0.05 significance level^23,24^.

### Standardized Precipitation Index (SPI)

We have also used the Standardized Precipitation Index (SPI)^25^ to measure the dry/wet conditions based on the IMD gridded monthly precipitation and CMIP5 simulations (using the same models as in the case of temperature). In this study, we opted for the 3-month scale SPI (SPI3) for detecting drought events and severe drought events, defined as SPI < − 1.0, and SPI < − 1.5, respectively.

Quantile regression analysis was used to investigate the relationship between heat waves and 3-month SPI (see Fig. 3). Quantile regression is a well-defined statistical framework for regression analysis on quantiles rather than the mean. Quantile regression aims to estimate conditional quantile values across a distribution function^26^. For a more detailed information on quantile regression the reader is referred to Ref.^27^.

## Supplementary Information

Supplementary Information.

## Data Availability

All data needed to evaluate the conclusions in the paper are present in the paper and/or the Supplementary Materials. Additional data related to this paper may be requested from the authors. The author would like to thank IMD for providing observational data and the GCM modeling groups, the Program for Climate Model Diagnosis and Inter-comparison (PCMDI), and the WCRP’s Working Group on Coupled Modeling for their roles in making available the WCRP CMIP5 multi-model datasets.
