# Supplementary material for: Anthropogenic influence on the changing risk of heat waves over India
Source: Sci Rep. 2022 Feb 28;12:3337. doi: 10.1038/s41598-022-07373-3 (PMC8885895; doi:10.1038/s41598-022-07373-3)
Supplement: Supplementary file 1 — Supplementary Information. [file 41598_2022_7373_MOESM1_ESM.docx]

**Anthropogenic influence on the changing risk of heat waves over India**

**P. Kishore^1^, Ghouse Basha^2*^, M. Venkat Ratnam^2^, Amir AghaKouchak^1, 3^, Qiaohong Sun**^3^, **Isabella Velicogna^1, 4^, and T.B.J.M. Ouarda^5^**

^1^Department of Earth System Science, University of California, Irvine, California, 92697, USA.

^2^National Atmospheric Research Laboratory, Gadanki, Tirupati, India.

^3^Department of Civil and Environmental Engineering, University of California, Irvine, California, 92697, USA.

^4^Jet Propulsion Laboratory, California Institute of Technology, Pasadena, CA 91109, USA.

^5^ INRS-ETE, National Institute of Scientific Research, Quebec City, Canada.

**Corresponding Author**

Dr. Ghouse Basha

National Atmospheric Research Laboratory

Department of Space, P.B. No123, Tirupath-517502, India.

Email: mdbasha@narl.gov.in

**SUPPLEMENTARY MATERIALS**


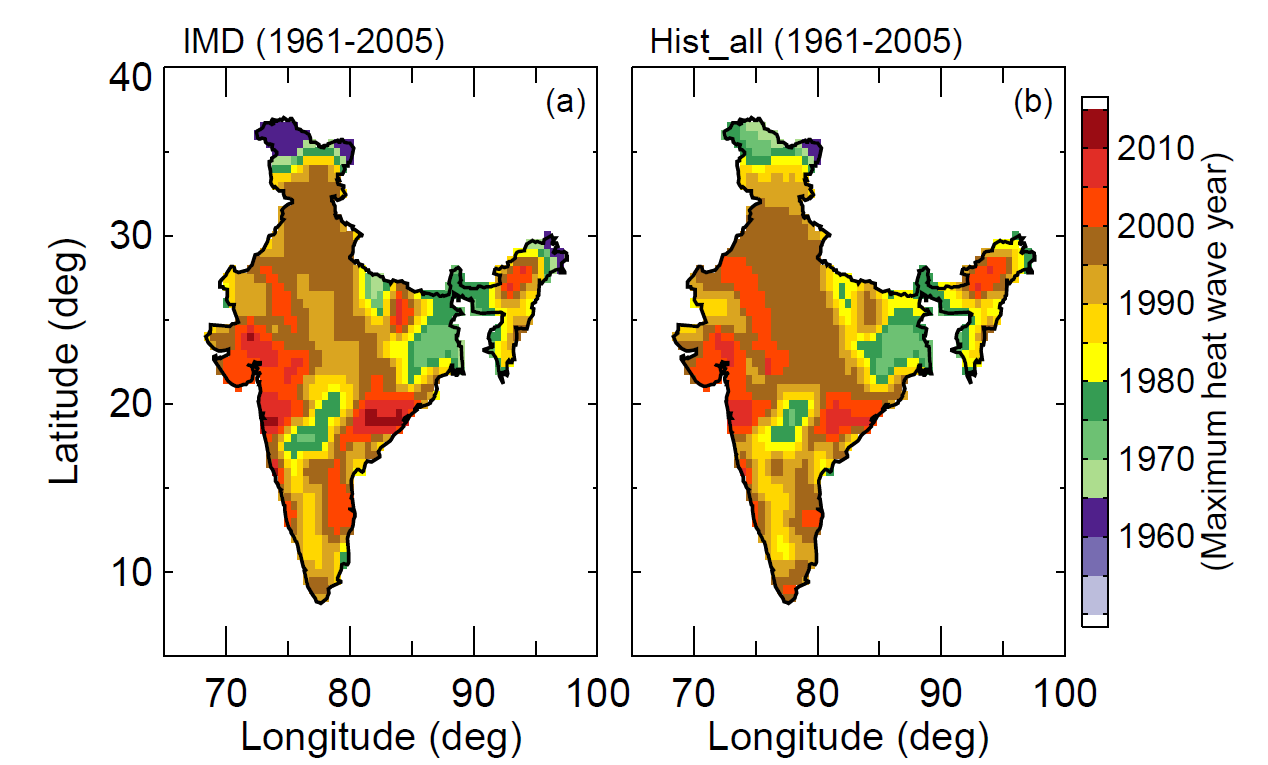


Figure S1. Year of occurrence of maximum heat wave based on the IMD data and historical simulations during 1961-2005.

**Hist HWMId and IMD SPI**

Figure S2. Quantile regression analysis between heat waves and 3-month standardized precipitation index (SPI03). Scatter plots of the maximum HWMId values for each grid location over India during the period 1961-2015 and the 3-month SPI + 1 month (a). The inset figure displays the spatial correlation maps of HWMId- SPI03. Panel (b) shows the corresponding regression slopes for 0.1-0.99 quantiles of the maximum HWMId-SPI03.


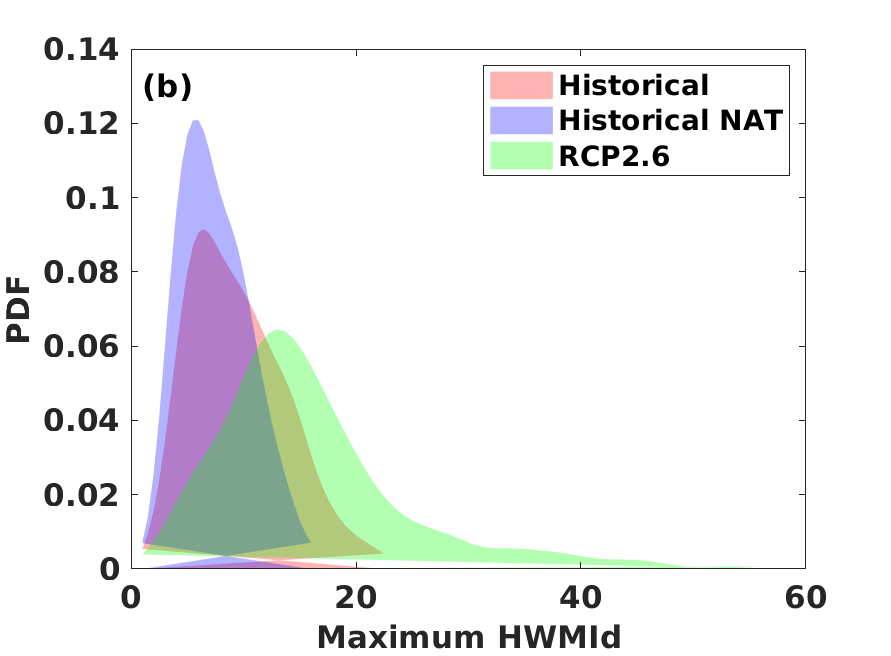

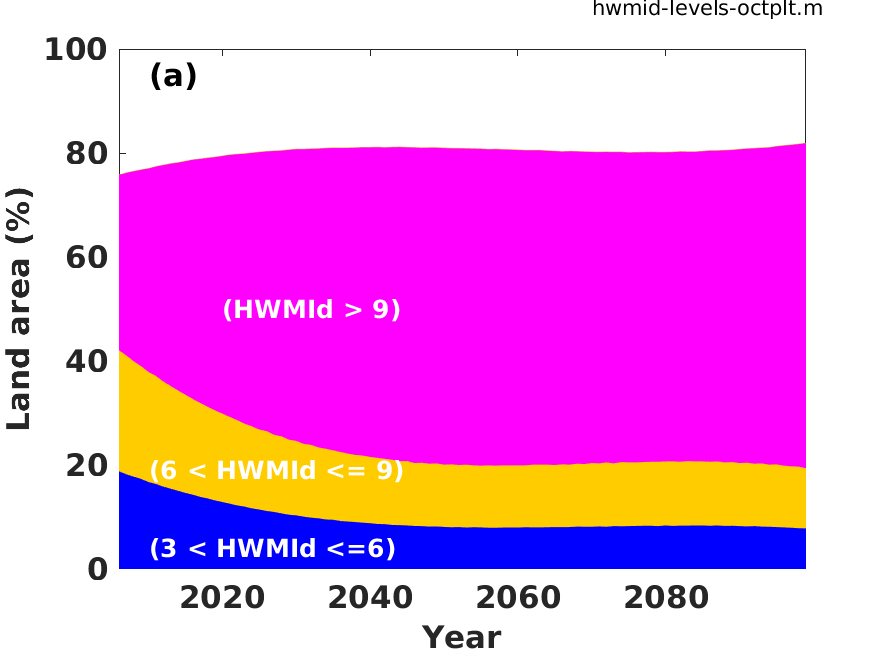


Figure S3: Same as figure 4 but for RCP2.6.


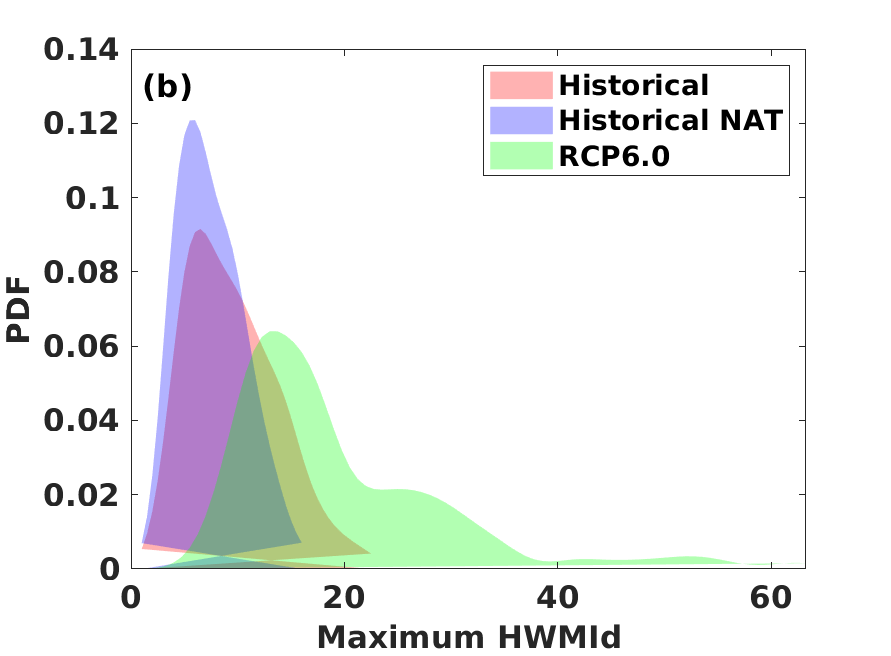

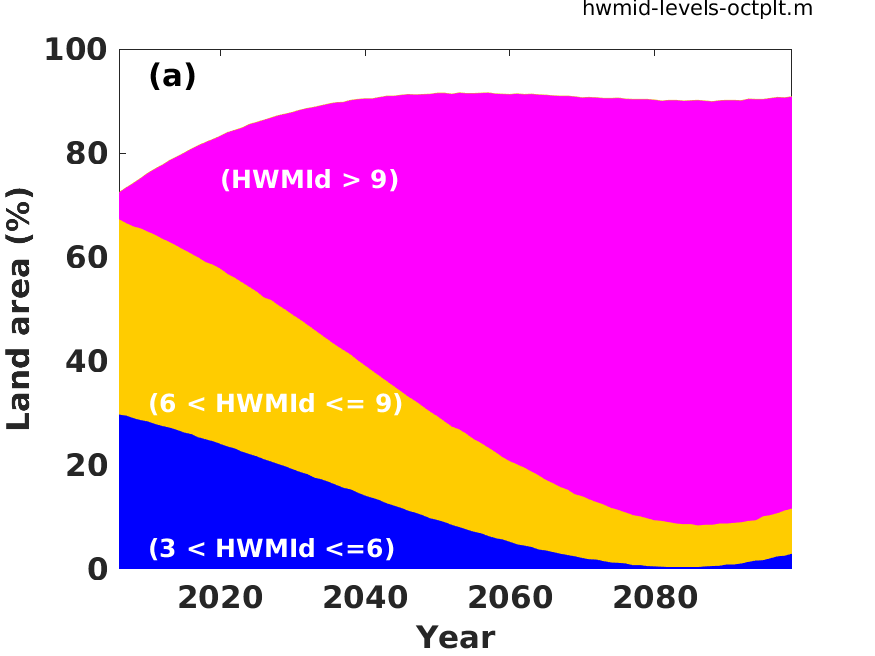


Figure S4: Same as figure 4 but for RCP 6.0.


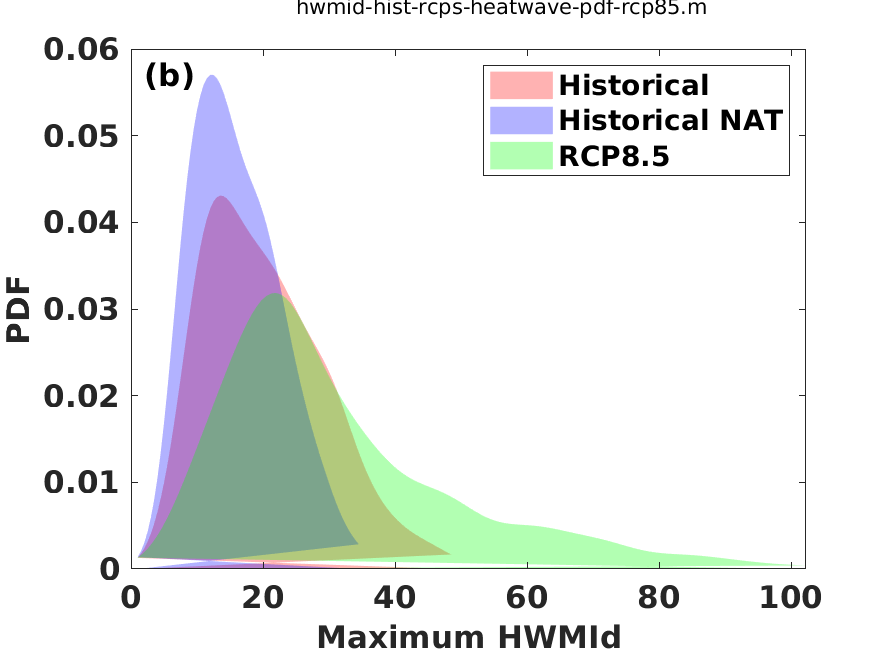

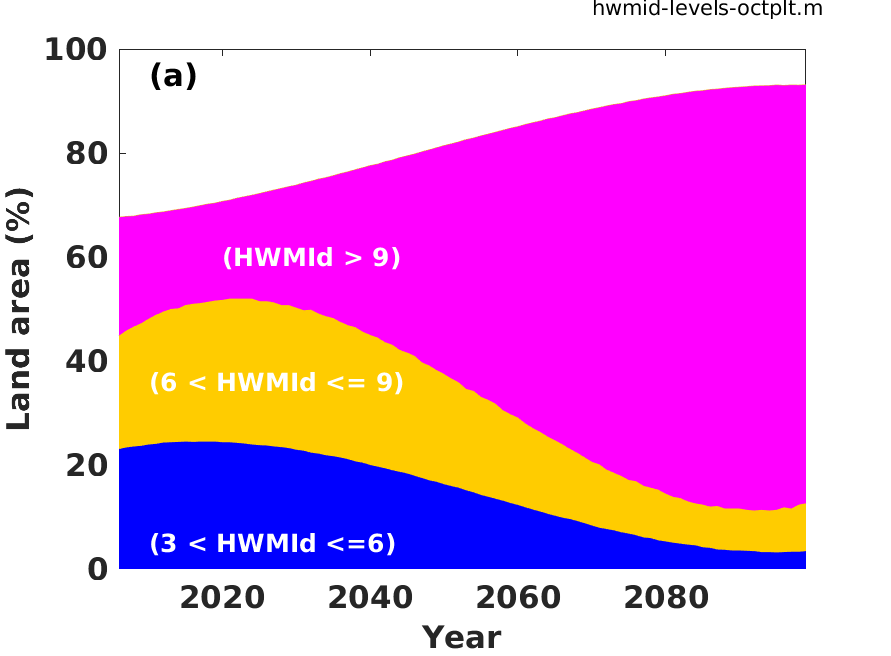


Figure S5. Same as figure 4 but for RCP8.5.


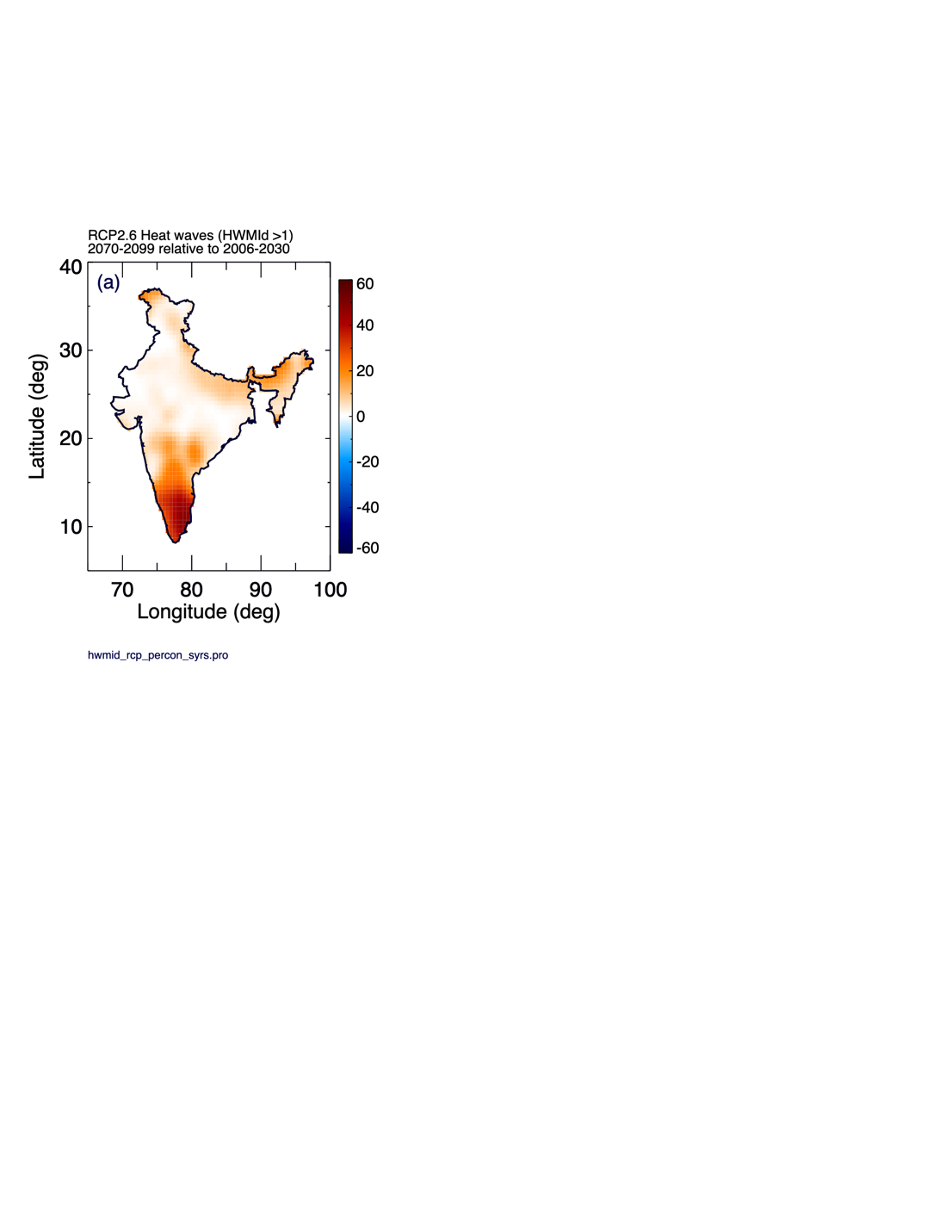

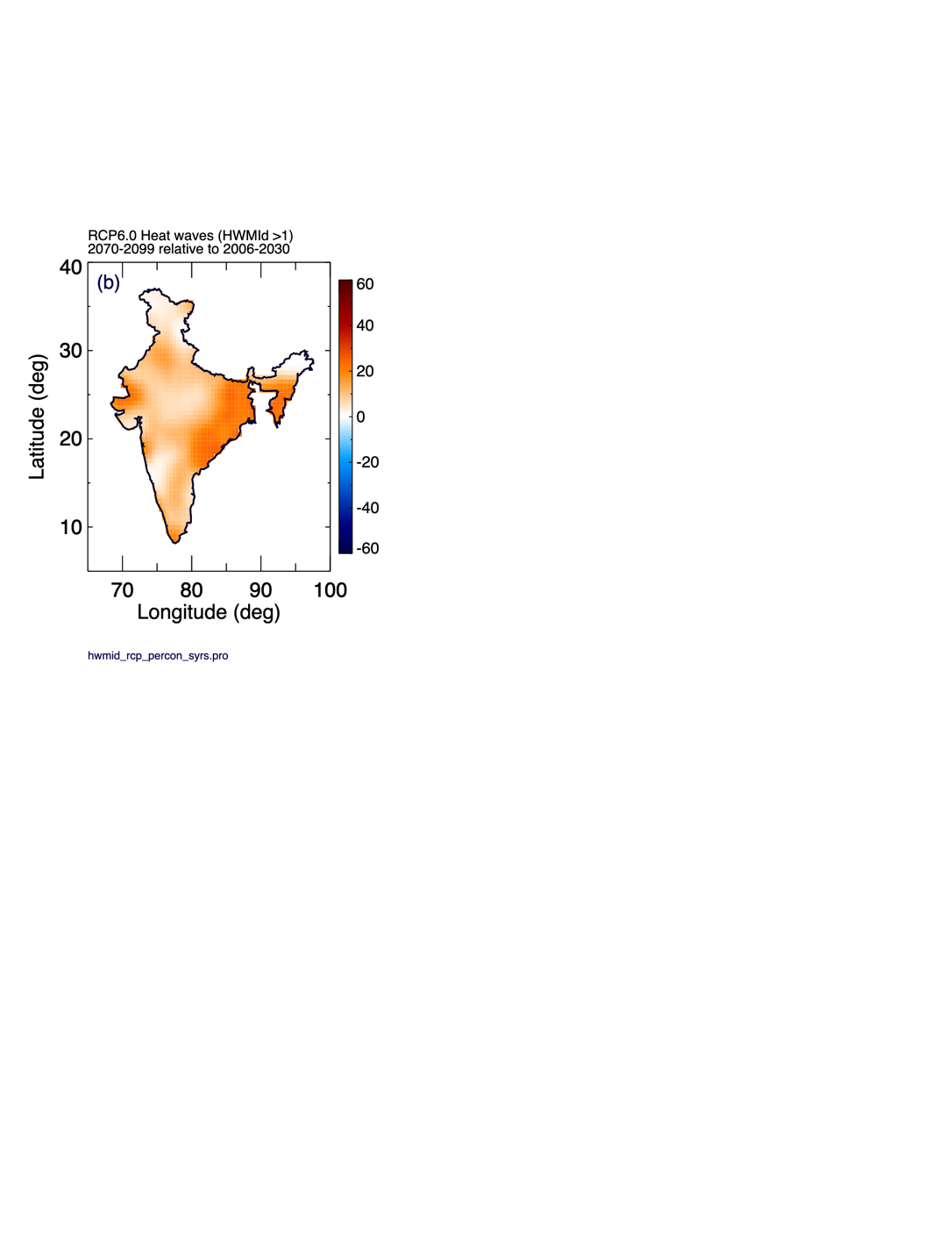

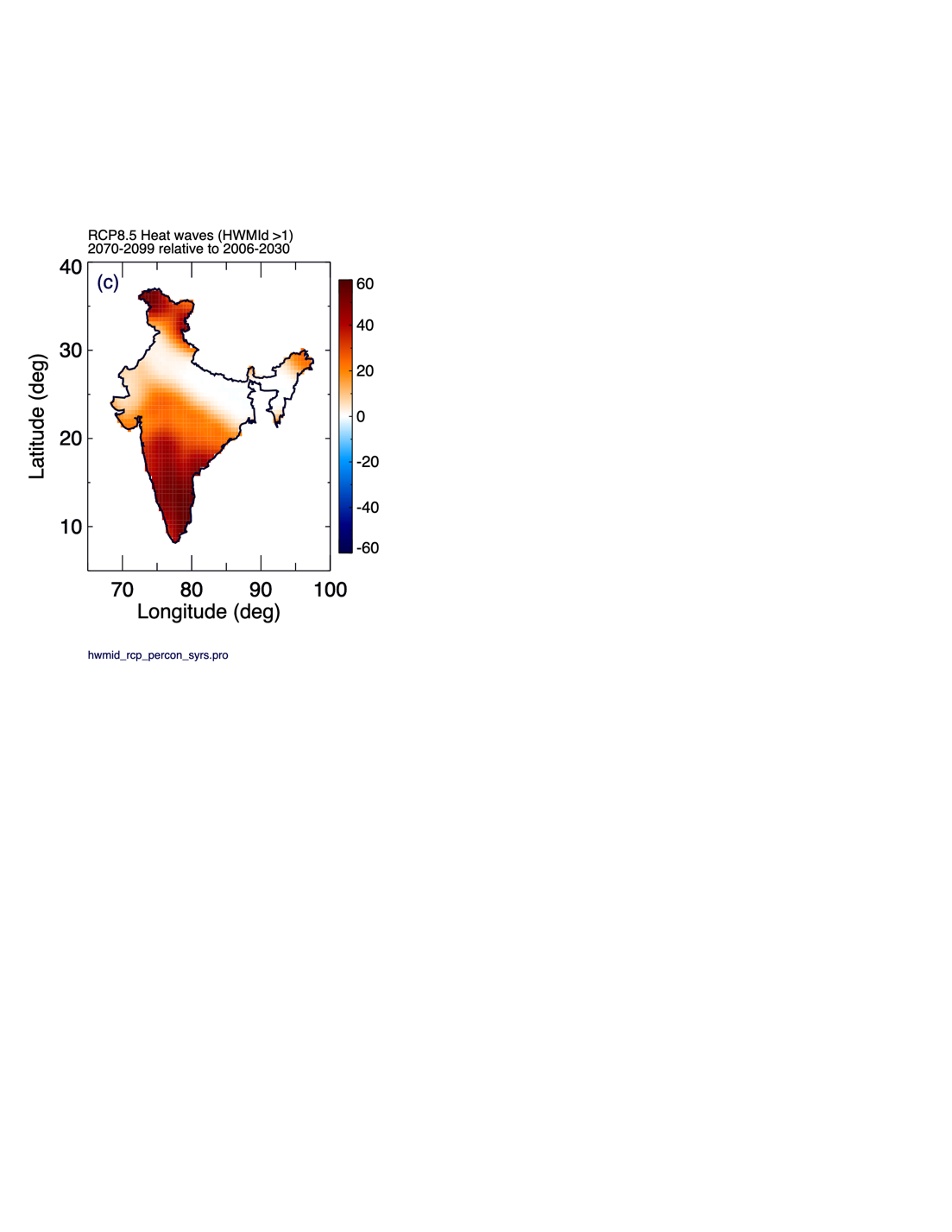


Figure S6. Spatial change in the frequency of concurrent heat waves (HWMId ≥1) and droughts (SPI≤-1) during 2070-2099 relative to 2006-2030. (a), (b), and (c) Heat waves occurring after preceding drought under RCP2.6, RCP6.0 and RCP8.5 scenarios. Heat waves occurring simultaneously with drought (d), (e) and (f) under RCP2.6, RCP6.0 and RCP8.5 scenarios.

| CMIP5 Data | | | | | | |
| --- | --- | --- | --- | --- | --- | --- |
| Model Name | NAT | Historical | RCP2.6 | RCP4.5 | RCP6.0 | RCP8.5 |
| CNRM-CM5 | Y | Y | Y | Y | Y | Y |
| CanESM2 | Y | Y | Y | Y | Y | Y |
| GFDL-CM3 | Y | Y | Y | Y | Y | Y |
| IPSL-CM5A-LR | Y | Y | Y | Y | Y | Y |
| MIROC5 | N | Y | Y | Y | Y | Y |
| MPI-ESM-LR | N | Y | Y | Y | Y | Y |
| NorESM1-M | Y | Y | Y | Y | Y | Y |
| bcc-csm1-1 | Y | Y | Y | Y | Y | Y |
| Total | 6 | 8 | 8 | 8 | 8 | 8 |

Table S1. List of CMIP5 models (Historical, NAT forcing and RCP’s) used in this study (Y indicates the forcing was used in this study and N indicates the simulation was not available). Model simulations were obtained from the CMIP5 website: <http://cmip-pcmdi.llnl.gov/index.html)>.
